# Prediction of antibiotic resistance from antibiotic susceptibility testing results from surveillance data using machine learning

**DOI:** 10.1038/s41598-025-14078-w

**Published:** 2025-08-20

**Authors:** Swetha Valavarasu, Yasaswini Sangu, Tanmaya Mahapatra

**Affiliations:** Department of Computer Science and Information Systems, Birla Institute of Technology and Science, Pilani, Pilani Campus, Vidya Vihar, Pilani, 333031 Rajasthan India

**Keywords:** Antibiotic resistance, Antibiotic susceptibility testing, Machine learning, AMR prediction, Antimicrobial resistance, Computer science

## Abstract

Antimicrobial resistance is a growing global health threat, and artificial intelligence offers a promising avenue for developing advanced tools to address this challenge. In this study, we applied various machine learning techniques to predict bacterial antibiotic resistance using the Pfizer ATLAS Antibiotics dataset. This comprehensive dataset includes patient demographic data, sample collection details, antibiotic susceptibility test results, and resistance phenotypes for 917,049 bacterial isolates. The dataset was divided into two subsets: Phenotype-Only and Phenotype + Genotype, excluding and including 589,998 isolates with genotype data, respectively. Both subsets underwent exploratory data analysis, preprocessing, machine learning model training, validation, and optimization. XGBoost consistently outperformed other models, achieving AUC values of 0.96 and 0.95 for the Phenotype-Only and Phenotype + Genotype sets, respectively. Hyperparameter tuning yielded slight accuracy improvements, while data balancing techniques notably increased recall. Across all models, the antibiotic used emerged as the most influential feature in predicting resistance outcomes. The SHAP summary plots generated provide insights into model interpretability. Our findings provide valuable insights into global AMR patterns and demonstrate the potential of AI-driven approaches for resistance prediction to help inform clinical decision-making and support the formulation of effective AMR mitigation policies, subject to the availability of highly granular datasets.

**Supplementary Information:**

The online version contains supplementary material available at 10.1038/s41598-025-14078-w.

## Introduction

Antimicrobial resistance (AMR) is a multiscale global public health threat that, if not addressed urgently, could lead to an estimated 39 million deaths in the next 25 years^1^. With a significant decline in the number of new antibiotics released in the market and a simultaneous increase in resistance to the ones currently in use, it is imperative that the emergence and spread of AMR be monitored and efforts be made to curb the issue. The different approaches taken to tackle the problem include monitoring molecular dynamics and genetic changes in pathogens using next-generation sequencing (NGS) data, collecting, and analyzing resistance patterns from antibiotic susceptibility testing (AST), developing new antibiotics and antimicrobial peptides (AMPs), drug combinations and adjuvants, and practicing antibiotic stewardship, among others^2^. These facets of data have also been used for machine learning (ML) applications^3^. Models have been built with high accuracy for developing new antibiotics and AMPs, drug combinations and adjuvants, analyzing antibiograms, enhancing AST methods, guiding antimicrobial therapy, analyzing NGS data to identify AMR genes for various pathogens, and predicting AMR to improve prediction-based diagnostics^4–7^.

Comprehensive AMR surveillance programs that monitor and assess ongoing changes in resistance to antimicrobial agents worldwide are administered by government bodies and funded by pharmaceutical industries in an effort to decrease morbidity and mortality caused by AMR in clinical settings by informing treatment guidelines and decisions, improving the effectiveness of intervention strategies, guiding national and international policies and antimicrobial stewardship programs, and directing the development of novel antimicrobial agents. Currently, numerous surveillance programs exist across different geographical locations to collect AMR data of varying scopes and types. Some of the notable ones include World Health Organization’s Global Antimicrobial Resistance and Use Surveillance System (GLASS)^8^, European Antimicrobial Resistance Surveillance Network, National Antimicrobial Resistance Monitoring System, English Surveillance Programme for Antimicrobial Utilisation and Resistance, Japan Nosocomial Infections Surveillance, One Health Trust’s ResistanceMap, Pfizer Antimicrobial Testing Leadership and Surveillance (ATLAS), and Merck Study for Monitoring Antimicrobial Resistance Trends (SMART)^9,10^. These programs primarily collect culture-based AST results (zone diameter or MIC values, which are then interpreted as susceptible, intermediate, or resistant according to international guidelines on clinical breakpoints) of representative clinical samples for various pathogen-antimicrobial agent pairs, with some programs like the Pfizer ATLAS collecting corresponding genotypic data as well for selective samples.

The conventional gold-standard method to determine antibiotic susceptibility to antibiotic agents and to identify AMR to treat infections is the in vitro laboratory phenotypic test^11^. However, this method is time-consuming and resource-intensive, especially for non-fastidious microbial pathogens and slow-growing bacteria like *Mycobacterium tuberculosis*. This makes it difficult and impractical to implement in actual clinical practice. An emerging alternative for detecting AMR is utilizing microbial genotype data instead of phenotype information, driven by advancements in sequencing technologies and lower sequencing costs. This genotypic approach offers faster AMR prediction compared to the traditional method by bypassing the need for culture-based laboratory procedures. Additionally, it provides insights into the genetic mechanisms responsible for AMR, enabling early detection of disease transmission^12^.

For machine learning-based AMR prediction, there have been studies conducted, majorly in high-resource settings, using clinical, epidemiological, and microbiome data such as demographic characteristics of patients, previous AST results, and prior antibiotic exposure data to predict drug resistance in infected patients, ML-assisted antibiotic prescription, and national and international AMR trends, among other applications^13^. Utilizing next-generation sequencing data, including whole genome sequencing, metagenomics, and transcriptomics data, AI/ML techniques aid in identifying resistance genes and mutations/variations to enable studying AMR development and support personalized treatment recommendations. With decreasing sequencing costs and expanding availability of genomic data, the information derived for AMR prediction and model interpretability is ever-expanding^14^. Predictive models for AMR aid in the provision of quick information for prompt AMR mitigation by finding hidden connections in data provided for monitoring AMR patterns using network analysis, time series analysis, and phylogenetic studies. These could help in the early detection of uncommon behaviors and clusters associated with drug resistance and overexpression of antibiotic-resistance genes, which in turn enables the implementation of disease transmission prevention efforts and understanding of the spread of resistance in hospitals and communities^4^.

In a review of machine learning prediction of AMR by Kherabi et al.^13^, nineteen out of the thirty-six studies included were based on previous AST results, with sample sizes ranging from a few hundred patients to a maximum of 68,472 patients. These studies were mostly localized to a particular ward, hospital, or country and were performed on a limited set of bacteria. On a global scale, in the study by Mutisya et al. 2024^15^, the prediction of AMR using machine learning was performed on the Pfizer ATLAS Antibiotics and Antifungal datasets with data collected from 2004 to 2021. This dataset has also been used to study resistance dynamics and resistance patterns across drug-bacterium pairs^16^. In this study, the Pfizer ATLAS Antibiotics dataset with data from 2006 to 19 was analyzed using a multivariable spatiotemporal analysis and found significant associations of AMR with country-level antibiotic sales for fluoroquinolone-resistant *E. coli* and *Pseudomonas*, as well as carbapenem-resistant *A. baumannii*. They also found a correlation between temperature and AMR in Enterobacteriaceae and with health system quality for all bug-drug combinations except for *Enterococci* and *Streptococcus pneumoniae*. Such studies showcase the utility of such robust surveillance data and help in understanding the status of AMR globally.

In our study, we have explored using machine learning techniques on the Pfizer ATLAS Antibiotics dataset for the prediction of antibiotic resistance to understand how to utilize such robust global surveillance datasets for real-world applications and the challenges encountered. In the process, we have performed exploratory data analysis, training, and testing of various machine learning models, explored methods for handling missing data and data imbalance, and assessed the models based on their performance metrics and optimized them. Feature importance and SHAP analysis were also done to identify which of the features had the most effect on resistance phenotype (susceptible, intermediate, resistant), the target variable, during classification by the model. The results obtained in this study could be a stepping stone for building tools to inform clinical practice and aid in policymaking for the surveillance of AMR on a global scale. This study expands the predictive framework to a globally diverse, multiclass (S/I/R) resistance phenotype using a robust surveillance dataset. Additionally, it explores the implications of both phenotype-only and genotype-inclusive models to evaluate the incremental predictive power of molecular features, along with providing insights into model interpretability.

## Results

### Exploratory data analysis

EDA was performed using matplotlib, pandas, and seaborn libraries to understand the underlying distributions and correlations in the dataset. The dataset has 917,049 rows with information on resistance interpretations of MIC values when tested against a panel of 50 antibiotic drugs for 324 bacterial pathogen species collected across 83 countries from 2004 to 2022, along with patient demographics. A subset of the dataset with 589,998 isolates contains data on the presence or absence of genotype markers such as CTXM, predominantly for ESBL isolates of the Enterobacteriaceae family.

The dataset contained the highest frequency of data collected from the United States of America with the majority share (31%), followed by Spain (12%), France (11.5%), Germany (10.9%), and China (6.1%), aligning with the notion that better monitoring resources are available in economically developed countries^17^. AMR is a looming threat in Sub-Saharan Africa^18^; however, there is a significant underrepresentation of data from this region. This disparity highlights the need for increased investments in AMR surveillance infrastructure in low- and middle-income countries (LMICs). Shown in Supplementary Figure S1 is the overall global distribution of antibiotic-resistant bacteria captured in the Pfizer ATLAS Antibiotics dataset.

**Fig. 1:**
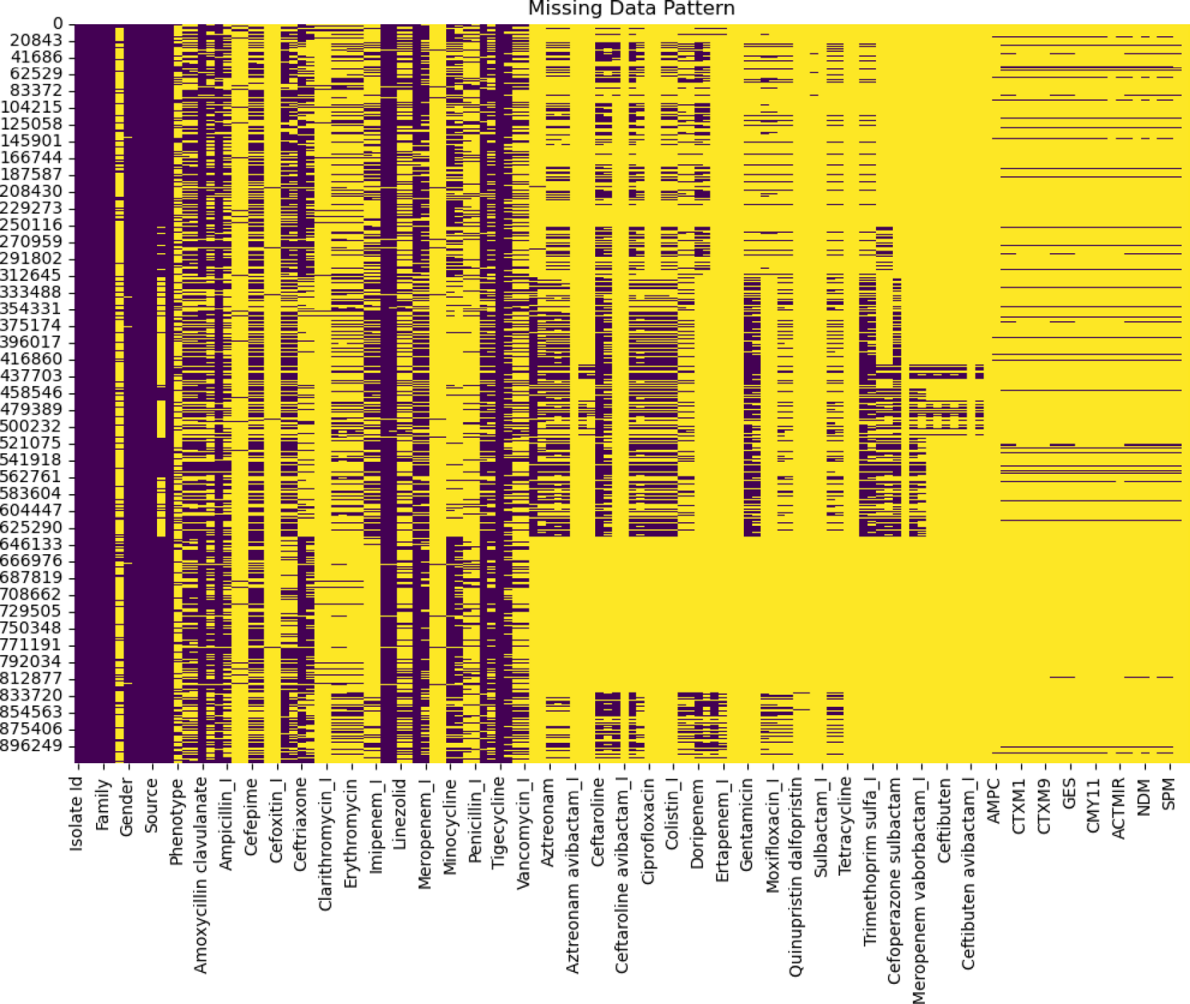
Missing data patterns in the dataset for each column are shown on the horizontal axis. Yellow represents missing data, and black represents available data.

Missing data is a major problem during prediction; a heatmap (shown in Fig. 1) was generated to understand the number of samples in each column where no data was available. In the heatmap, yellow indicates the absence of data, while purple indicates the presence. No data was missing for the initial columns, such as Isolate ID, Family, and Gender. In contrast, a major part of the data was absent for genetic markers like AMPC, CTXM1, etc., which is reasonable given the extensive resources required for genetic marker analysis in routine surveillance systems. This visualization highlights the need for methods to handle missing data while building machine learning models for practical applications. Imputation is a powerful technique for effectively handling missing data in machine learning, as used by Mutisya et al. 2024^15^ in their study to predict AMR from surveillance data. Although it increases the prediction accuracy of the models, from a clinical perspective, it needs to be done with caution as it could mislead decision-making processes if assessment of the imputation methods is not done^19^.

**Fig. 2:**
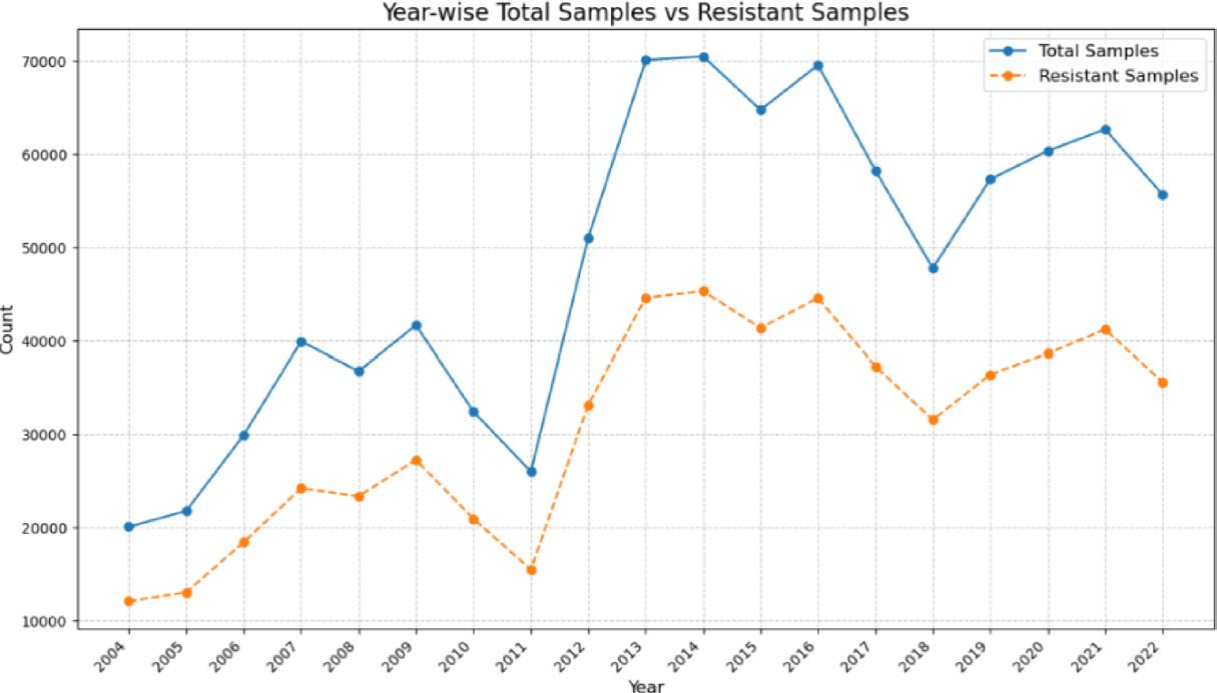
Year-wise Total Samples vs. Resistant Samples from 2004 to 2022.

Temporal analysis was performed to further understand the year-wise distribution of total samples and resistant samples between the years 2004 and 2022. The total number of samples showed a significant increase up to 2014, reflecting better surveillance and data collection efforts over time. However, a minor decline in total samples can be observed after 2016. The resistance rate can be deduced visually by comparing the two-line plots (Fig. 2). If the resistant line grows at a similar rate to the total line, it implies that the proportion of resistant cases remains largely unchanged, indicating persistent resistance. If the gap between the total and resistant samples increases, it may indicate effective interventions or reduced resistance levels. In contrast, if the lines converge, it could signal an increase in resistance rates. The trend in resistant samples mainly follows the trend of total samples, suggesting a persistent antimicrobial resistance. This could also occur due to sampling bias.

Correlation analysis was conducted on the full dataset, including genotypic features indicating the presence or absence of β-lactamase genes such as TEM, AMPC, and NDM^20,21^. These genes are particularly relevant in ESBL-producing organisms like *Escherichia coli* and *Klebsiella* spp., where CTX-M, SHV, and TEM serve as hallmark markers^22^. Co-occurrence of AmpC and carbapenemase genes (e.g., NDM, OXA, IMP) suggests extensive multidrug resistance, limiting therapeutic options^23^.

**Fig. 3:**
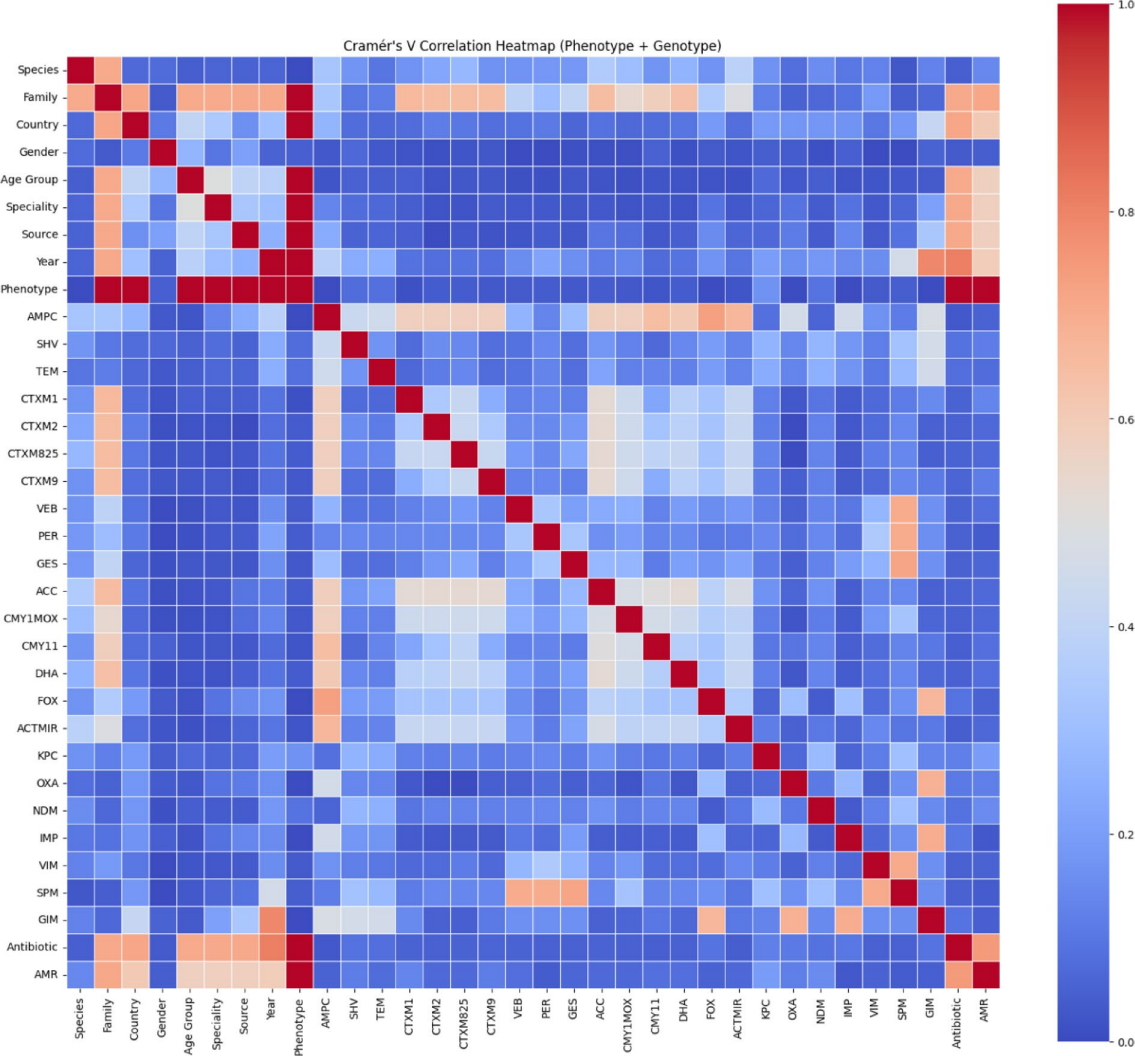
Cramer’s V correlation plot for all features in the dataset.

To examine associations between bacterial, demographic, genotypic, and phenotypic variables, a Cramér’s V correlation heatmap was generated (Fig. 3). This method, suited for categorical data, revealed strong correlations between species and family, and moderate-to-strong associations between key resistance genes and bacterial taxa (e.g., CTX-M1, SHV, TEM). Carbapenemase genes such as NDM and VIM also showed moderate associations with specific species, indicating species-driven gene dissemination patterns^24^.

Antibiotic susceptibility patterns were moderately correlated with AMR outcomes, validating the phenotypic influence of tested antibiotics. In contrast, demographic features like age group, gender, and country exhibited weak correlations with genotypic resistance or AMR phenotype. However, the phenotypic resistance class (e.g., MRSA) is predictive of whether the isolate is Susceptible, Intermediate, or Resistant to an antibiotic, with high correlation values. This reflects biological causality, as phenotypes directly influence susceptibility. Source, Species, and Family also show moderate correlations with Phenotype and AMR. This implies that ecological or biological niches (e.g., certain sources like urine, blood, or respiratory) and taxonomic identity affect resistance patterns.

Exploratory analysis of year and country data showed minimal to moderate associations with pathogen and antibiotic variables. This suggests some geographic and temporal variation in testing or prevalence, but not strong enough to drive stratified modeling in this study. These findings support the inclusion of genetic features for improved model interpretability and reinforce the need for global, standardized surveillance of resistance trends.

### Model performance

The dataset was pre-processed and was followed by training and testing classification models for the prediction of antibiotic resistance into Susceptible, Intermediate, and Resistant categories. Phenotype-Only and Phenotype + Genotype datasets were derived from the original dataset and processed individually to avoid selection bias since only a subset of the dataset contains genotype data. The models trained included simplistic models like Logistic regression as well as complex ensemble models like XGBoost. The model performance metrics for the initial set of models with all features included and implemented are given in Table 1 for the Phenotype-only and Phenotype + Genotype datasets. Simpler models like logistic regression and KNN have lower accuracies, whereas more complex models like XGBoost and Gradient Boost have better prediction ability and higher scores on all metrics. For the Phenotype-only dataset, SVM has the highest precision. For both datasets, XGBoost was the best-performing model, with its AUC being the highest (95%) and its accuracy slightly enhanced with hyperparameter tuning and ten-fold cross-validation. Supplementary Table S2 shows the parameters used for model development and the tuning parameters used for hyperparameter tuning.

**Table 1:** Model performance metrics for Phenotype-Only and Phenotype + Genotype datasets.

Model	Phenotype-Only					Phenotype + Genotype				
	F-1 score	Precision	Recall	Accuracy	ROC-AUC	F-1 score	Precision	Recall	Accuracy	ROC-AUC
Logistic Regression	0.89	0.85	0.68	0.89	0.94	0.82	0.86	0.72	0.82	0.92
Random Forest Classifier	0.89	0.78	0.71	0.89	0.93	0.84	0.82	0.76	0.84	0.94
XGBoost	0.90	0.84	0.70	0.90	0.96	0.85	0.85	0.76	0.85	0.95
AdaBoost	0.89	0.86	0.67	0.89	0.86	0.81	0.84	0.71	0.81	0.94
Gradient Boost	0.89	0.87	0.68	0.89	0.94	0.83	0.87	0.72	0.83	0.93
SVM	0.90	0.88	0.69	0.90	0.87	0.84	0.84	0.74	0.84	0.93
KNN (k = 100)	0.89	0.87	0.67	0.89	0.94	0.76	0.83	0.62	0.76	0.89
Naive Bayes (Bernoulli)	0.77	0.59	0.65	0.77	0.90	0.77	0.78	0.68	0.77	0.89
Modifications done on the XGBoost model:										
Undersampling	0.83	0.66	0.80	0.83	0.83	0.80	0.74	0.78	0.80	0.80
Oversampling (SMOTENC)	0.86	0.68	0.78	0.86	0.86	0.85	0.79	0.78	0.85	0.85
Bayesian Optimization	0.91	0.83	0.71	0.91	0.91	0.86	0.83	0.78	0.86	0.86
Cross Validation	0.90	0.84	0.71	0.90	0.90	0.86	0.83	0.78	0.86	0.86
Bayesian optimization + Cross Validation	0.91	0.84	0.71	0.91	0.91	0.85	0.83	0.77	0.85	0.85

**Fig. 4:**
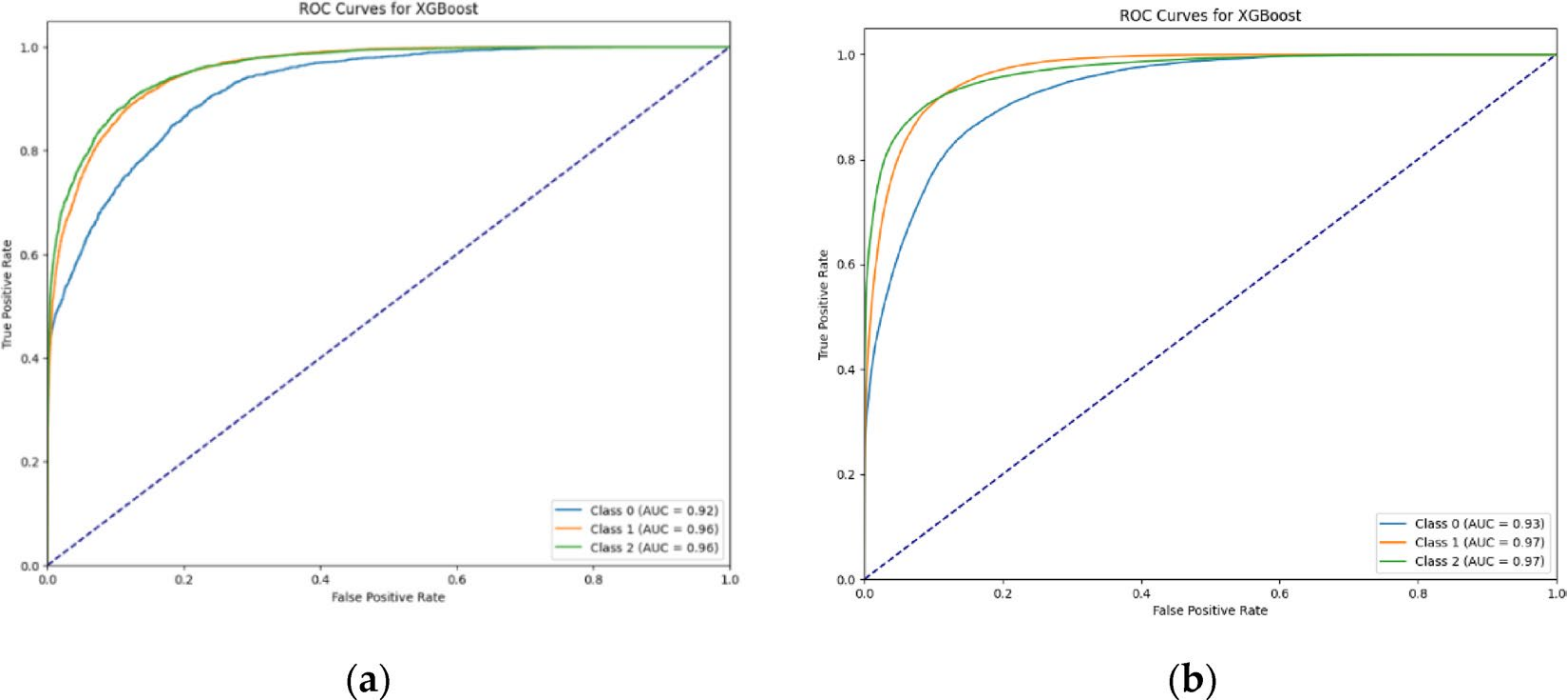
ROC-AUC curves for XGBoost models for (**a**) Phenotype-Only and (**b**) Phenotype + Genotype datasets. Classes 0,1, and 2 are Intermediate, Resistant, and Susceptible, respectively.

Shown in Fig. 4 are the ROC-AUC curves of the XGBoost models. ROC-AUC measures the model’s ability to distinguish between classes. A higher AUC indicates a better-performing model, with 1.0 being the best possible score and 0.5 representing random guessing. To handle the multi-class nature of the problem, the One-vs-Rest (OvR) strategy was implemented. For each class, the model was trained to distinguish it from the others, and binary classification was performed on each one. The ROC curve was plotted by calculating the False Positive Rate (FPR) and True Positive Rate (TPR) for each class. The AUC for each class was then calculated, with a higher AUC indicating a better performance at distinguishing that class from the others. To statistically evaluate differences in model performance, the DeLong test was applied to compare the AUC values between models. The results indicated no statistically significant difference in AUC values, suggesting that the models exhibit similar discriminatory power. Additionally, the bootstrapped confidence intervals around the AUC estimates were narrow, indicating stability and low variability in the AUC across resampled datasets. While this supports the robustness of the models’ performance, it also highlights the limitation of relying solely on AUC for model selection. Given the lack of significant difference in AUC, additional metrics—such as precision, recall, and F1-score—as well as model interpretability, were considered to enable a more comprehensive comparison of model behavior and utility.

### Data balancing

**Fig. 5:**
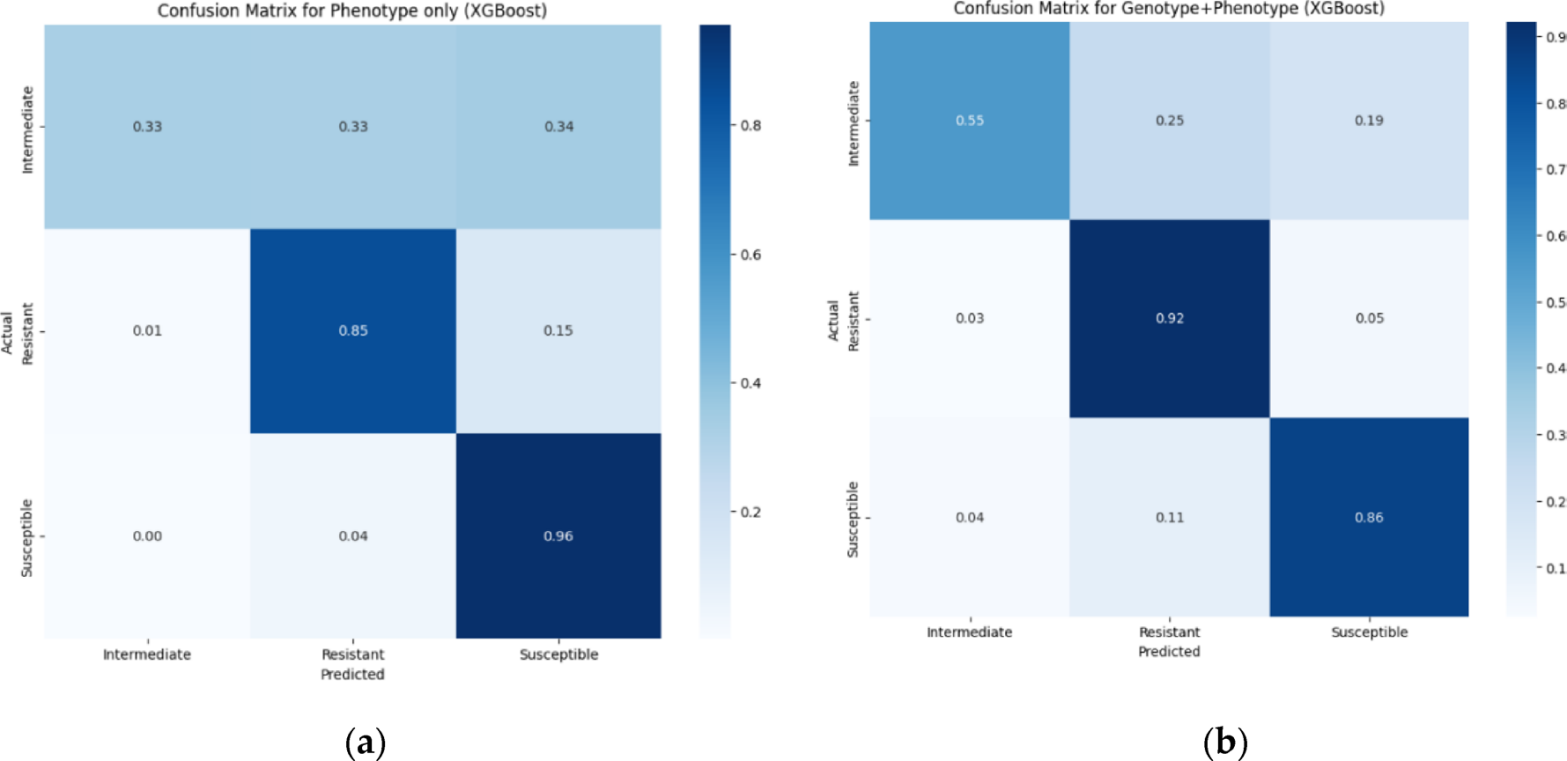
Confusion matrices of the XGBoost models for (**a**) Phenotype-Only and (**b**) Phenotype + Genotype datasets.

The accuracy of prediction is lowest for the Intermediate category for both Phenotype-only and Phenotype + Genotype datasets, as seen in the confusion matrices of the XGBoost models (Fig. 5). This is because of data imbalance, with the number of intermediate samples being considerably lower compared to resistant and susceptible samples, which could affect the model’s ability to generalize effectively (S/I/R data distribution shown in Supplementary Figure S2). Imbalanced datasets could cause ML models to bias toward the majority class, misclassify rare but clinically important classes, and underperform in metrics like recall and F1-score for minority classes. Data balancing using under-sampling of susceptible samples and oversampling of intermediate samples done on the XGBoost models improved recall.

### Feature importance

**Fig. 6:**
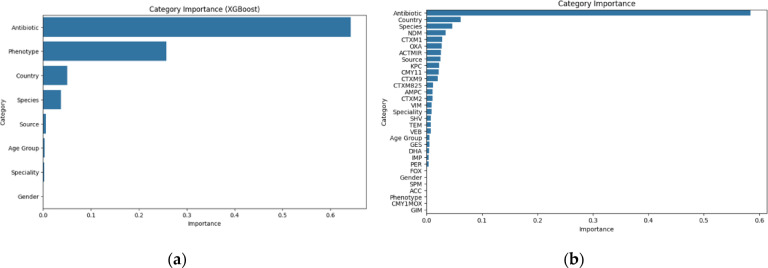
Feature importance plots of XGBoost models for (**a**) Phenotype-Only and (**b**) Phenotype + Genotype datasets.

For models like Logistic Regression, Random Forest, Gradient Boosting, AdaBoost, and XGBoost that support feature analysis, the corresponding scores that indicate how much each feature contributes to the model’s performance were extracted. These feature importance scores reflect how often and how effectively a feature contributes to the decision-making process in the model’s trees. These scores were used to determine which groups of features had the most significant impact on the model’s predictions (Fig. 6).

### SHAP (SHapley additive exPlanations) analysis

**Fig. 7:**
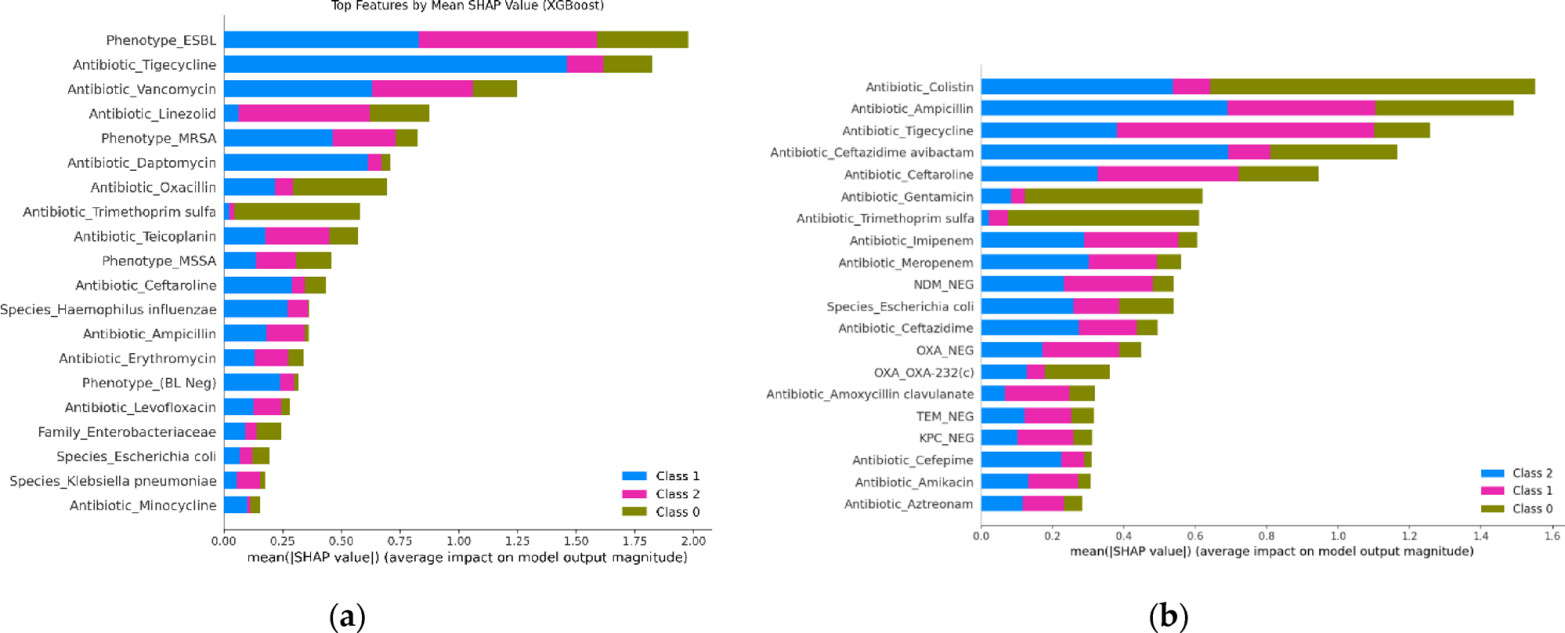
SHAP plots of XGBoost models for (**a**) Phenotype-Only and (**b**) Phenotype + Genotype datasets, where classes 0, 1 and 2 are Intermediate, Resistant and Susceptible, respectively. SHAP summary plot illustrating the contribution of each feature to the model’s predictions. Features are ranked by importance (y-axis), and the horizontal distribution shows their impact on the prediction output.

SHAP analysis^25^ for the XGBoost model trained on the Phenotype-only dataset (summary plot shown in Fig. 7) revealed that individual features such as Phenotype_ESBL, Phenotype_MRSA, and antibiotics like tigecycline, vancomycin, and oxacillin were among the most influential in predicting AMR phenotypes. These features are known resistance indicators and reflect the model’s reliance on clinically relevant patterns^26–30^. Most top features contributed to the prediction of the intermediate class, while fewer features drove predictions for susceptible, suggesting class-wise differences in feature influence. The SHAP plot enhances interpretability by identifying which phenotypic or treatment features the model used most heavily, supporting transparency and clinical relevance.

The SHAP summary plot for the Phenotype + Genotype dataset (Fig. 7) provides a global view of the most influential features driving model predictions for antimicrobial resistance phenotypes—Susceptible (Class 2), Resistant (Class 1), and Intermediate (Class 0). Features such as Antibiotic_Colistin, Antibiotic_Ampicillin, and Antibiotic_Tigecycline exhibited the highest average SHAP values, indicating strong contributions to the model’s predictive accuracy. Notably, Colistin and Ampicillin showed the greatest impact on predicting the Intermediate phenotype (Class 0), as seen by the dominance of green bars^31,32^. Conversely, features like Ceftazidime avibactam, Cefaroline, and resistance genes such as OXA_NEG and NDM_NEG contributed significantly to identifying Resistant phenotypes (Class 1), suggesting the model correctly leveraged key molecular determinants of resistance^24,33^. Susceptible predictions (Class 2) were more associated with features such as *Escherichia coli*, Cefepime, and absence of resistance genes (KPC_NEG, TEM_NEG), indicating that the model identified typical patterns associated with non-resistant strains^34,35^. These findings not only validate the model’s behavior but also enhance interpretability by aligning with known biological mechanisms. Such model explanations are essential in AMR contexts where understanding the why behind a prediction is as important as the prediction itself. Although genotype features offered modest overall performance gains, they showed disproportionately high importance in ESBL and carbapenem resistance cases. This underscores the clinical value of including molecular features in high-priority resistance scenarios.

## Discussion

Machine learning models were implemented using the Pfizer ATLAS Antibiotics surveillance dataset for the prediction of antibiotic resistance in bacteria within the dataset. Our models predict AMR for single drug-bug combinations and currently do not account for multiple drug resistance. Tree-based models performed better than simpler models^14^, in capturing the resistance patterns from global data for an antibiotic-bacteria pair. The best-performing model for prediction was XGBoost, in agreement with previous studies^15^, which was further optimized using feature selection and hyperparameter tuning to improve its accuracy. While prior studies emphasized predictive performance, we contribute to interpretability by integrating SHAP interaction values and comparative model explainability across different data subtypes. This allows more meaningful biological insights, especially in ESBL-producing organisms.

Antibiotics are the most critical predictor in the AMR prediction models, which aligns with the current research that antibiotic exposure creates selective pressure, leading to resistance development^36^. Different antibiotics have varying resistance impacts, reflected in multidrug resistance patterns across bacterial species. Models that utilize antibiotic data perform better as they capture these subtle antibiotic-bacteria interactions^37^. Geographical variations in AMR are well-documented. Regional factors such as antibiotic stewardship policies, socioeconomic conditions, and antibiotic consumption trends influence resistance patterns^36,37^. However, local resistance trends cannot be captured if data is not available at least at a state level, especially for huge countries like India with resistance patterns varying in different states^38^. Genetic markers have moderate but essential importance in predicting AMR. For our best-performing model, XGBoost, NDM, CTXM1, and OXA, were present in the top five features. Current research supports this, as NDM (New Delhi Metallo-*β*-lactamase) is known to produce enzymes that degrade carbapenem antibiotics^39^. CTXM1, part of the CTX-M β-lactamase family, confers resistance to third-generation cephalosporins and is commonly found in *E. coli* and *K. pneumoniae*^40^. OXA-*β*- lactamases, like OXA-48, are also known to provide resistance to both penicillins and carbapenems^41^. Studies show that machine learning models benefit from incorporating genetic marker data, especially when predicting resistance across different bacterial strains. Models like Gradient Boosting and XGBoost capture these interactions due to their ability to handle non-linear relationships^42^. Feature importance is model-specific, and the skew in the raw dataset towards ESBL-producing Enterobacteriaceae could have impacted the output. Feature importance reflects how much a variable contributes to a model’s predictions, and varies across algorithms. In our dataset, the overrepresentation of ESBL-producing *Enterobacteriaceae* may bias the model to emphasize features associated with these organisms, limiting generalizability. To address this, we applied resampling and caution that the reported importance scores are influenced by this underlying data skew.

Demographic features like age and gender have minimal influence on AMR prediction models for data from across the world, although they have been shown to have an effect at the national level^43^. While these factors may play a role in infection risk, they do not significantly impact resistance development, as resistance is driven primarily by antibiotic exposure and microbial genetics^44^. The above analysis aligns with findings that AMR dynamics are better explained by antibiotic usage patterns and pathogen-specific factors rather than patient demographics^37^.

There were also some model-specific differences observed in feature importance. Gradient Boosting and XGBoost outperform simpler models like Logistic Regression in capturing complex, non-linear interactions among features. This explains why genetic markers show slightly higher importance in these models^45^. Logistic Regression, although simpler, still highlights antibiotics as extremely dominant, reflecting the direct relationship between antibiotic exposure and resistance development. The use of absolute coefficients in Logistic Regression also offers good interpretability, which complements the predictive power of tree-based models^42^.

The outcome of any resistance prediction study is as good as the input data, which currently has many caveats and shortcomings, to which the models’ prediction accuracies could be attributed. These stem from noise, biases, and anomalies from discrepancies in AST protocols, resistance phenotype interpretation guidelines, MIC breakpoints, data imbalance with significantly more susceptible phenotypes, as well as a bias towards high-income countries^16^. The antibiotic used was the most important feature in AMR prediction, while demographic factors like age and gender did not seem to have a significant effect on the model’s prediction; however, these factors play a major role in determining the socioeconomic effects of AMR. This study shows the potential and challenges of utilizing current AMR surveillance datasets in machine learning applications. Handling missing data is a commendable task in extending this study to build tools for clinical applications and predict resistance trends. We acknowledge that AST methods vary by region, and the dataset’s overrepresentation of certain geographies (e.g., USA, Spain) and susceptible phenotypes may limit generalizability. Our mitigation strategy included data resampling and separate phenotype/genotype models. However, standardized global sampling and AST protocols would greatly enhance model robustness and global applicability.

The predictions could be made with more confidence if genomic sequencing data was available for all the isolates, with the possibility of correlating the AST results with AMR genes^46^. The availability of these gene sequences could provide critical information on drug-bacteria interaction, which cannot be inferred with just the presence or absence of the genes. Apart from this, AMR surveillance datasets from other industry monitoring systems could be integrated with the Pfizer ATLAS dataset to increase the size of the dataset and its scope for AMR prediction while incorporating checks for differences in methodologies. The models can also be extended to surveillance data from government initiatives and international agencies like the WHO GLASS data to make the most of existing data to predict future AMR patterns across the world^47^. Also, agencies collecting AMR surveillance data from AST test results could consider including more specific details of patient age, location, and time of sample collection, particularly for being able to implement accurate and usable machine learning models such as time-series forecasting models to predict future trends^48^, for clinical use and formulate strategies for global and country-level policy-making to curb AMR. Uniformity in guidelines for AST protocols for AMR surveillance data collection, interpretation of its results, and more representation of isolates from LMICs could immensely enhance the usability and harmonization of these datasets at a global scale for accurate antibiotic resistance predictions using interpretable machine learning models for building future-ready prediction tools. Future work should involve validation on independent datasets—such as those from WHO GLASS or regional health authorities—to assess real-world clinical applicability. Prospective evaluation in clinical decision support systems is needed before deployment. Expanding the input dataset by incorporating data from other AMR surveillance programs and additional genetic features could help increase the model’s robustness and generalizability. Prediction of multidrug resistance is another avenue for exploration.

The application of machine learning to surveillance data for AMR prediction raises important ethical considerations related to data governance, patient anonymity, and responsible model deployment. Although the dataset used in this study was fully de-identified and collected under established surveillance protocols, minimizing privacy risks, clinical translation of such models must be approached with caution. Specifically, there is a risk of algorithmic bias arising from uneven geographic or demographic representation, particularly the underrepresentation of LMICs or certain resistance mechanisms. To ensure fair and effective use, machine learning models should be integrated with local epidemiological data and antimicrobial stewardship frameworks. Interpretability tools such as SHAP improve model transparency, but deployment in real-world clinical settings must also involve thorough local validation, regulatory oversight, and context-specific adaptation.

## Methods

The Pfizer ATLAS Antibiotics dataset, one of the many industry monitoring systems for AMR surveillance available on request from the Vivli AMR Register (https://amr.vivli.org)^49^, containing patient data along with extensive metadata, was acquired. The dataset contains MIC values from antibiotic susceptibility assays and their interpretations based on Clinical and Laboratory Standards Institute (CLSI) standards for 917,049 bacterial isolates from 83 countries collected over the years 2004 to 2022.

The dataset contains patient demographic data, details of bacterial pathogen isolate collection, MIC values obtained from an in vitro drug susceptibility assay, and resistance interpretations done in accordance with international standards. Exploratory data analysis and the machine learning workflow were performed using Python version 3.8.

The raw dataset was preprocessed to ensure compatibility with machine learning workflows. Missing values were replaced with the placeholder ‘NA’ where appropriate, particularly in columns containing MIC values and their corresponding AMR interpretations. These missing entries were not imputed, as their absence typically reflects that susceptibility testing was not performed for the specific antibiotic–bacterium combination, rather than random omission or measurement error. Consequently, such entries were excluded during downstream processing. This approach avoids introducing artificial values into critical microbiological fields, thereby preserving the integrity of the resistance phenotype representation. Initially, the columns Study and Isolate ID were removed as they do not have any effect on the resistance patterns. The column State had values available only for the United States of America and was omitted, considering the other 82 countries did not. The raw MIC values were removed, and the data was transformed for each of the rows to contain the antibiotic tested and its corresponding resistance phenotype interpretation since, in the original dataset, the antibiotic susceptibility results were spread across multiple columns for each of the antibiotics tested.

A subset of the dataset with 589,998 isolates had genotypic data, primarily in Enterobacteriaceae isolates with ESBL phenotype, which was made into a separate dataset named Phenotype + Genotype. After transformation, the original dataset was considered separately after the exclusion of genotypic data to eliminate selection bias and named Phenotype-Only. Thus, two distinct datasets were used to implement two distinct sets of machine-learning models, including Logistic Regression, Random Forest Classifier, Naïve Bayes (Bernoulli), K-Nearest Neighbors (KNN), Support Vector Machine (SVM), Adaptive Boosting (AdaBoost), Gradient Boost, and Extreme Gradient Boosting (XGBoost), with an 80:20 train-test split, to maintain a clean train-test split to simulate unseen data. The models classify and predict the probability that the bacterial isolate is susceptible/intermediate/resistant to a particular antibiotic, given the other patient demographics and clinical features. The models were implemented using various libraries - scikit-learn for traditional machine learning algorithms, XGBoost for extreme gradient boosting, and cuML, a RAPIDS library, for GPU implementations of SVM and KNN. All machine learning models were initially trained using the default parameter settings of their respective libraries (e.g., scikit-learn v1.4.0 and XGBoost v1.7.6). A detailed list of the parameter values used for each algorithm is provided in Supplementary Table *S1*. This includes key parameters such as max_depth, learning_rate, and subsample (for XGBoost), even when default values were retained, to ensure clarity and reproducibility. Model metrics, such as accuracy, precision, F1 score, AUC, and recall, were compared. Model interpretability was evaluated using both feature importance and SHAP summary plots. These provide insight into feature behavior across resistance classes and are essential for policy translation. Among the models trained, the best-performing model, XGBoost, was chosen for further optimizations. The number of susceptible samples was disproportionately higher than resistant and intermediate samples, whereas intermediate samples had very low representation. To balance the data, under-sampling of susceptible samples and oversampling of intermediate samples, using SMOTENC (Synthetic Minority Over-sampling Technique for Nominal and Continuous variables), was done, and the model metrics of these two models were observed. Hyperparameter tuning was performed using the Optuna library, which implements Bayesian optimization to efficiently explore the hyperparameter space. Both standalone and combined strategies involving hyperparameter tuning and k-fold cross-validation (k = 10) were employed during model training. The use of 10-fold cross-validation ensured robust model evaluation by reducing the risk of overfitting and provided a more generalized performance estimate across different subsets of the data. This approach also introduced controlled randomization into the model training process, addressing potential bias associated with a fixed 80:20 train-test split.

Based on the results obtained in the initial models, a final set of best-performing models using XGBoost was constructed. From the feature importance charts and lack of any direct effect on the resistance profiles, the features Isolate ID, Study, State, Gender, Age group, and In/Outpatient were removed from the datasets. Hyperparameter tuning was done to improve performance, and the model’s performance was assessed.

This study exclusively used de-identified surveillance data publicly available from the Pfizer ATLAS database, ensuring no personal identifiers or protected health information were involved. The ethical use of machine learning models in healthcare settings necessitates transparency, fairness, and responsible implementation. Throughout our model development and evaluation, we adhered to principles outlined in internationally recognized frameworks such as the WHO’s guidance on the ethics and governance of AI for health (2021)^50^ and the OECD AI Principles^51^. Particular attention was given to mitigating algorithmic bias through resampling techniques and ensuring interpretability using SHAP. Although no formal ethics approval was required for this secondary data analysis, the broader implications of clinical deployment are acknowledged and discussed in the Discussion section.

## Conclusions

In this study, we developed machine learning models to predict antimicrobial resistance using surveillance data from the Pfizer ATLAS Antibiotics dataset. The models were evaluated and compared based on their predictive performance using various evaluation metrics. We have also attempted to address the issue of data imbalance using data balancing techniques. Feature importance and SHAP analysis were analyzed for the best-performing XGBoost models to gain insights into key predictive variables. Additionally, we have discussed the challenges and opportunities associated with utilizing large-scale surveillance datasets and outlined future directions, including the incorporation of more granular data and the integration of multiple surveillance sources to enhance predictive accuracy. Although this study does not directly integrate into clinical workflows, it lays a foundation for risk-stratification tools and AMR trend analysis in surveillance contexts. This could support treatment guideline revisions or antimicrobial stewardship policy development, after being subjected to prospective validation. These models highlight variables that could inform surveillance and empirical therapy in settings with similar pathogen/antibiotic profiles.

## Supplementary Information

Below is the link to the electronic supplementary material.

Supplementary Material 1

## Data Availability

This publication is based on research using data from Pfizer obtained through the Vivli AMR Register https://amr.vivli.org. The Pfizer ATLAS Antibiotics dataset (https://searchamr.vivli.org/datasetDetails/fromSearch/ce7367e1-c91a-4de1-87e4-6e4d8f834085) used in this study is available on request from the Vivli AMR Register. Data requests should be directed to the Vivli AMR platform and are subject to the data-sharing agreements and policies set by the platform. For further details regarding the data request process, refer to the official documentation of the Vivli AMR Register or contact their support team at amr@vivli.org. The source code of the implementation is available at https://github.com/softwareinnovationslabBITS/AMR_AST_ML.
