# Supplementary material for: Prediction of antibiotic resistance from antibiotic susceptibility testing results from surveillance data using machine learning
Source: Sci Rep. 2025 Aug 20;15:30509. doi: 10.1038/s41598-025-14078-w (PMC12368220; doi:10.1038/s41598-025-14078-w)
Supplement: Supplementary file 1 — Supplementary Material 1 [file 41598_2025_14078_MOESM1_ESM.pdf]

Prediction of antibiotic resistance from antibiotic susceptibility testing results from surveillance data using machine learning

Swetha Valavarasu [1], Yasaswini Sangu [1] and Tanmaya Mahapatra [1*]

**Model**	**Key Parameters**
Logistic Regression	C=1.0, penalty='l2', solver='lbfgs', max_iter=1000, random_state=42
Random Forest	n_estimators=100, criterion='gini', max_features='sqrt', bootstrap=True, random_state=42
XGBoost	objective='binary:logistic', device='cuda', eval_metric='mlogloss', tree_method='hist', max_depth=6, n_estimators=100, random_state=42
AdaBoost	n_estimators=50, learning_rate=1.0, random_state=42
Gradient Boosting	n_estimators=100, learning_rate=0.1, loss='log_loss', max_depth=3, criterion='friedman_mse', random_state=42
Linear SVM (SGD)	loss='hinge', penalty='l2', alpha=0.0001, max_iter=1000, tol=0.001, random_state=42
KNN (k=100)	n_neighbors=100, metric='minkowski', p=2, weights='uniform'
Bernoulli Naive Bayes	alpha=1.0, binarize=0.0, fit_prior=True

Supplementary Table S1: A detailed list of key parameter values for each algorithm. For all the models, the default parameter settings of their respective libraries were used.

Tuning parameters	List of values	Best parameters
max_depth	4,10	9
n_estimators	100, 500	384
learning_rate	0.001, 0.3	0.14
subsample	0.6, 1.0	0.8
colsample_bytree	0.6, 1.0	0.9

Supplementary Table S2: Tuning parameters considered in Bayesian Optimization

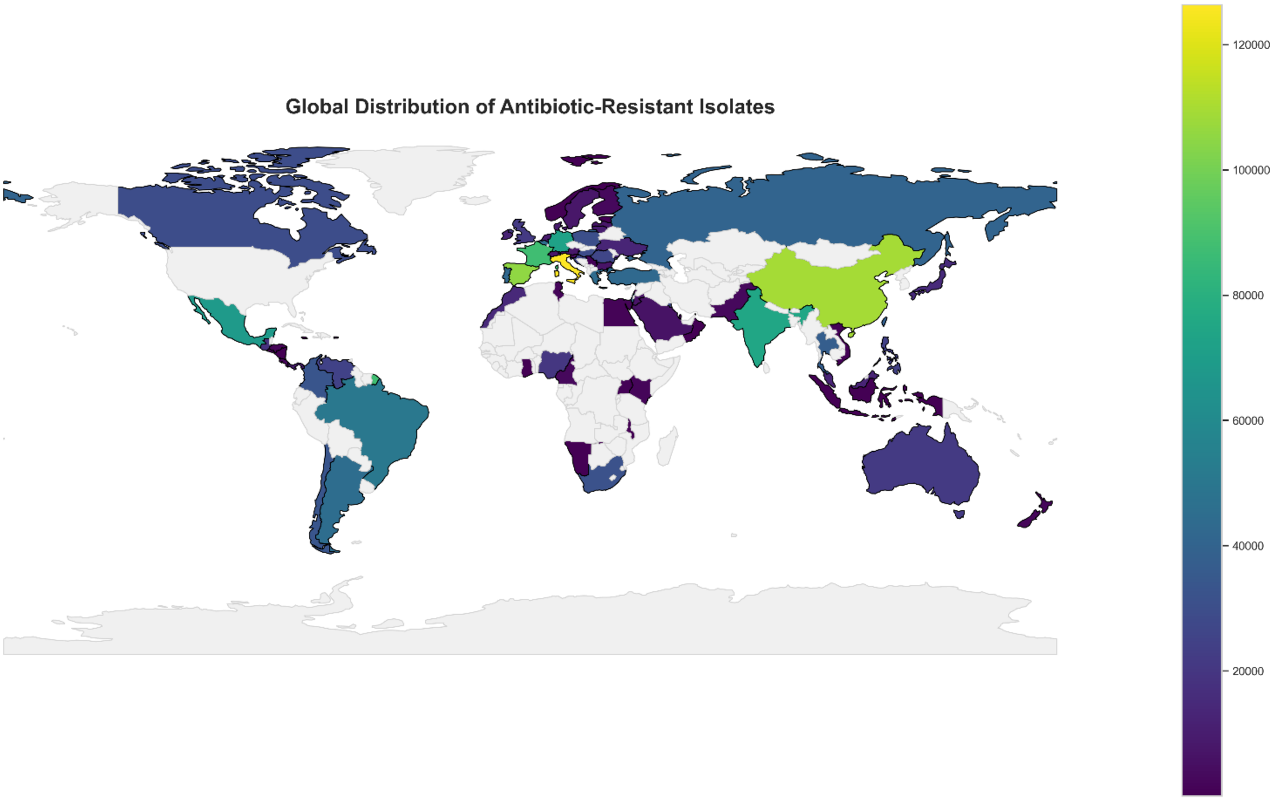

Supplementary Figure S1: Global distribution of resistant samples as per the Pfizer ATLAS Antibiotics dataset from the years 2004 to 2022.

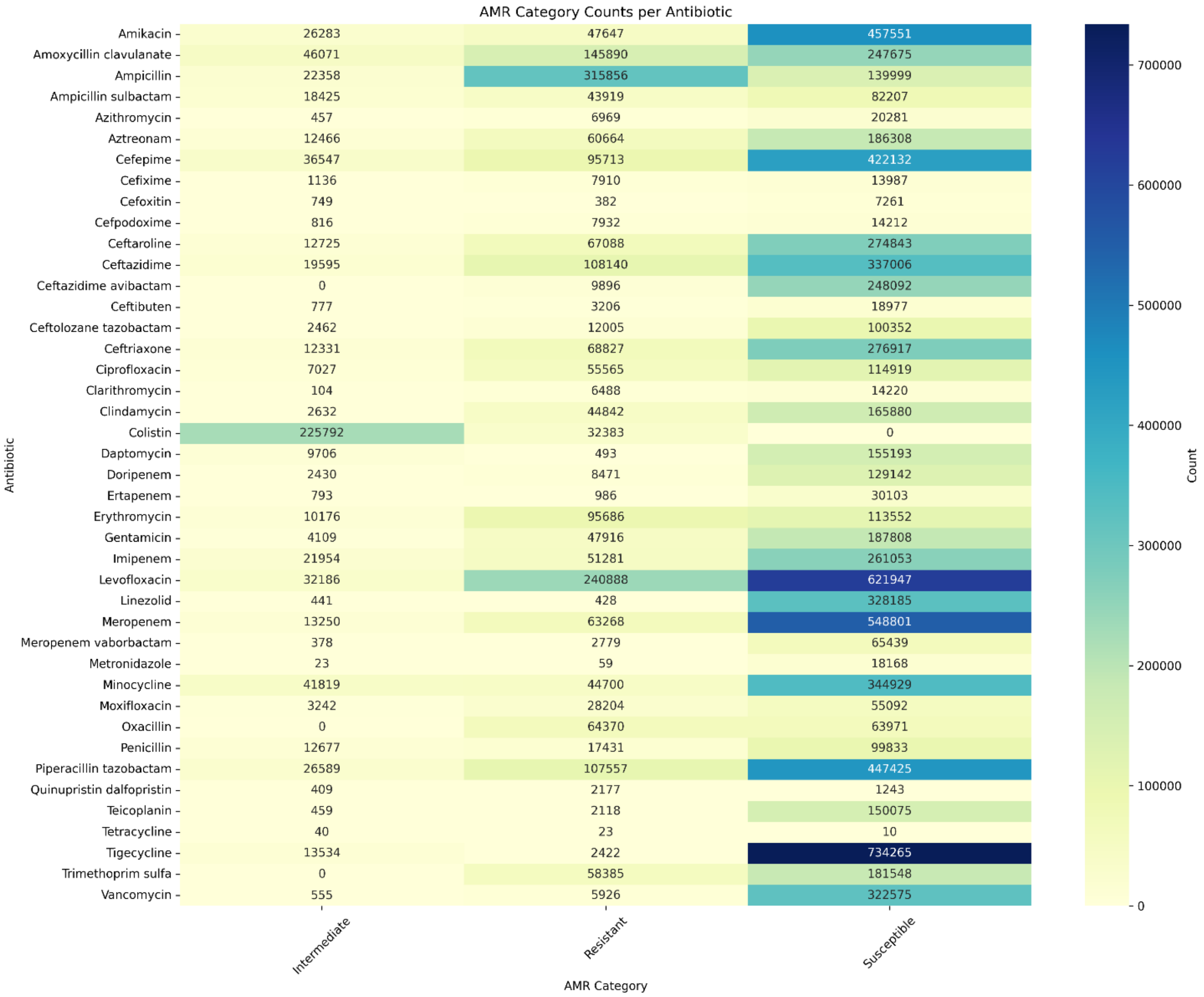

Supplementary Figure S2: Heatmap of AMR category counts per antibiotic. The imbalance in the dataset can be seen here with the disproportionately high number of Susceptible samples when compared to the underrepresented Intermediate category
